# Brain mineralocorticoid and glucocorticoid receptor balance in neuroendocrine regulation and stress-related psychiatric etiopathologies

**DOI:** 10.1016/j.coemr.2022.100352

**Published:** 2022-06

**Authors:** Edo Ronald de Kloet

**Affiliations:** Division of Endocrinology, Department of Medicine, Leiden University Medical Center, University of Leiden, Leiden, the Netherlands

**Keywords:** Glucocorticoid receptors, Mineralocorticoid receptors, Brain, Stress, Coping, Adaptation, Cognition, Emotion, Neuroendocrine regulation

## Abstract

Cortisol and corticosterone (CORT) coordinate circadian events and manage the stress response by differential activation of two complementary brain receptor systems, i.e., the mineralocorticoid receptor (MR) and the glucocorticoid receptor (GR), which mediate rapid non-genomic and slow genomic actions. Several recent discoveries are highlighted from molecular fine-tuning of the MR/GR balance by FKBP5 to CORTs role in neural network regulation underlying stress adaptation in emotional, cognitive, and social domains of behavior. The data suggest that MR mediates CORT action on risk assessment, social interaction, and response selection, while GR activation promotes memory consolidation and behavioral adaptation; there are also sex differences in CORT action. New evidence suggests that targeting the MR/GR balance resets a dysregulated stress response system and promotes resilience.

“*Endocrinology is a concept, an approach, or even can be considered a method. Whatever the specific endocrine subdiscipline, topic or subject might be, the binding element is the objective, which is the understanding how signals coordinate the processes in cells, tissues and organs.*” 1

## Introduction

Today, the above statement about endocrinology is still as current as it used to be. It certainly applies to this contribution, which is about the neuroendocrinology of cortisol and corticosterone (collectively called CORT) from gene to behavior. CORT is best known for its catabolic action in energy metabolism and the regulation of inflammatory and immune processes. Textbook knowledge is also that CORT coordinates circadian and sleep-related events [1] as well as the response to stressors with the goal to promote resilience and adaptation by feedback on the brain [2]. Beyond these “activational” actions, CORT also has “organizational” effects in brain development: CORT can program an emotional brain for life during the experience of adversity, trauma, and neglect in perinatal life [3]. CORT is also a significant factor in the aging process and can affect resilience to neurodegenerative diseases and psychiatric disorders [4, 5, 6].

To better understand the role of CORT in health and disease, Hans Selye distinguished already more than 70 years ago the action of antagonistic adaptive hormones (pro-inflammatory mineralocorticoids and anti-inflammatory glucocorticoids) which can set disease susceptibility at different levels, i.e., the “*pendulum hypothesis*” [7]. Today, this opposite action, exerted by two apparent “antagonistic” hormones, can be captured by CORT only, because the stress hormone activates in a complementary manner mineralocorticoid receptors (MR, encoded by the *Nr3c2* gene) and glucocorticoid receptors (GR, encoded by *Nr3c1*) [8,9]. Box 1 briefly summarizes the evolutionary aspects of MR and GR [10] and its implications.

Box 1 **Evolutionary significance of coordinated MR and GR-mediated actions**

MR and GR were duplicated from a common ancestor gene some 450 million years ago, first appearing in cartilaginous fishes [10]. The ancestral function was largely conserved in the MR lineage up to mankind. Hence, mammalian MR is still promiscuous in binding besides CORT, also aldosterone, deoxycorticosterone, and the sex steroid progesterone. Aldosterone only appeared in lungfish and lobe-finned fish, which are forerunners of terrestrial amphibians, reptiles, birds, and mammals. Because of CORT inactivation by 11-HSD2, aldosterone is the principal ligand for MR in epithelial cells involved in the regulation of electrolyte balance. In contrast to MR, the GR further gained during evolution selectivity to CORT. Interestingly, the evolutionary studies do not account yet for the membrane-associated MR and GR.

The properties and cellular localization of the receptors have been leading for studies into the MR/GR concept and its underlying action mechanism. It is a big leap, however, from molecular changes at the membrane level or in the genome toward a higher level of integration important for cellular homeostasis and excitability or on the systems level for physiological regulations and behavioral adaptation. To better appreciate its complexity, the reader is referred to an overview article by Sapolsky et al., 2000 that examines the physiology of permissive, stimulatory, suppressive, and preparatory actions of CORT using a variety of endocrine, pharmacological, and genomic approaches [4]. This analysis has been further elaborated over the years, for instance, in cardiovascular health [90,91], adipose metabolism [92], skin [93], immune system, and microglia's [94, 95, 96] and of course the neural networks in the brain. In all these tissues, MR and GR are cellularly co-localized and complement each other’s functions.

For integration at a higher level of complexity, the brain is of course extremely important since circuits that govern alarm reactions, appraisal processes, and response selection have the capacity to direct in *top-down* fashion the individual's behavioral adaptation. It is perhaps therefore no surprise that “uncertainty in predicting outcome” or “frustration with not receiving an expected reward” are among the most severe “psychological” stressors. Control of such stressors relies on information processing in limbic-forebrain circuitry, which is richly endowed with MR and GR. This limbic-forebrain processing of information operates in complex feedforward and feedback relationships, with CORT acting in *bottom-up* fashion to coordinate energy allocation in the brain with the body's performance [59].

In a recent review, questions were raised about the concept of the complementary MR and GR-mediated actions in stress and adaptation [15]. For instance, (i) is the balance in MR and GR-mediated actions to maintain homeostasis and support allostasis not a too simple concept? (ii) Does the MR/GR balance account for the enormous diversity in CORT action? (iii) What is the role of MR and GR in long-term early life programming effects of CORT? (iv) Which ratio of MR vs GR-mediated actions is favorable for resilience? And what about the effect of genetic polymorphisms? (v) more recently, what is the implication of the observation that some genomic binding sites for MR become saturated with higher concentrations of CORT than required for CORT binding to the MR itself? Is MR-GR heterodimerization involved in the larger MR capacity of the genomic actions, or is actually receptor turnover rate limiting? (vi) And what about the rapid membrane-associated vs the slow genomic MR and GR-mediated actions [13]?

Notwithstanding all these questions, genomic MR is activated already at very low basal CORT concentrations, thus fulfilling the criteria for at least permissive actions of CORT, while genomic GR only becomes activated after acute stressors and at the circadian peak. But, during ultradian rhythmicity, the transient GR activation is important for CORT responsivity, and the circadian rise in CORT-GR helps to prepare for upcoming daily activities, while stress-induced GR activation prevents the primary defense mechanisms from overshooting. However, if stress persists and the individual fails to cope, CORT turns into a proverbial “death signal” through dysregulated MR and GR.

Alt-text: Box 1

MR and GR are “complementary” because under basal a.m. conditions low levels of CORT occupy mostly the high-affinity MR, while during the circadian rise both MR and GR are activated. During the stress response, the lower affinity GR becomes progressively occupied over the 30–60 min after stressor experience. Obviously, the context in which the MR and GR operate in reaction to (or anticipation of) a stressor is entirely different from that during the circadian variation, when the hormone acts in preparation of daily activities and sleep-related events. Furthermore, besides the high-affinity CORT—MR in non-epithelial cells, there is also an aldosterone—selective MR in epithelial cells that is engaged in the regulation of electrolyte homeostasis [11]. MR and GR mediate in addition to the genomic actions of CORT and also rapid non-genomic actions via putative membrane-associated receptors [12,13]. MR and GR are co-localized in numerous tissues and occur with a very high expression in the limbic-cortical brain regions that are engaged in emotional and cognitive aspects of coping with stress and behavioral adaptation [5] (Box 2)**.**

Box 2 **MR and GR properties**

CORT binds with a tenfold lower affinity to GR than to MR, which implies that GR becomes activated only with rising hormone levels after stress and during circadian and ultradian peaks [15]. MR can be aldosterone-selective as in epithelial cells engaged in the regulation of electrolyte homeostasis and neurons of the n. tractus solitarii and circumventricular organs involved in salt appetite because of the intracellular breakdown of CORT by 11β-OH steroid-dehydrogenase type 2 (11-HSD2) [11]. The CORT-preferring MR occurs in non-epithelial cells expressing the type 1 isoform 11-HSD1, notably in the limbic-forebrain regions where MR is abundantly co-expressed with GR [97]. Because of its high affinity to CORT, the MR is retained in the nucleus over the ultradian cycle suggesting that for MR the receptor properties are rate limiting rather than the ligand concentration as is the case for GR but see Refs. [18,19]. MR and GR mediate not only the slow CORT action on gene expression but also operate as non-genomic mediators in mitochondria [85,98] or are associated with membranes regulating the release probability of excitatory glutamate via MR or inhibitory endocannabinoids via GR. These membrane-associated MR and GR have lower affinity to CORT and can rapidly respond to changes in circulating CORT concentration [99,100].

Alt-text: Box 2

The studies over the past decades led to the concept that “*upon imbalance in MR-GR regulated limbic-cortical signaling pathways the initiation and termination of the stress response is compromised. This may lead to a condition of HPA axis dysregulation and impaired behavioral adaptation, which can enhance susceptibility to stress-related neurodegeneration and mental disorders*” [5,14,15].

In this contribution, recent discoveries in the neuroendocrinology of CORT action are reported. Thus, Sections Fine-tuning MR:GR balance with FKBP5, Novel Hippocampal MR phenotype, Novel MR gene targets contain new data on molecular aspects of MR and GR-mediated actions. Sections GR and the hippocampal memory engram, Salience and executive networks, mPFC projectome, coping and neuroendocrine control are about novel aspects of MR and GR function in circuits underlying the processing of information from the perception of a stimulus to stress adaptation. Sections CORT patterns, Reset of the stress response system are on the role of MR and GR in CORT secretory patterns, including sex differences in CORT action, and how a dysregulated CORT action can be reset. This contribution is concluded with a perspective on how the new knowledge on CORT neuroendocrinology may contribute to a better understanding of resilience to psychopathology. The themed issue formula demands a concise presentation of new developments only.

## Fine-tuning MR/GR balance with FKBP5

MR- and GR-hsp90 are part of a distinct multimeric protein complex. Once CORT binds to the receptors, the complex dissociates allowing the steroid receptor to associate with specific chromatin sites and DNA sequences. The multiprotein complex includes the co-chaperone FK506-binding protein 51 (FKBP5) encoded by the *Fkbp5* gene. FKBP5 is induced by GR activation during stress and subsequently this protein decreases GR binding to CORT. Accordingly, stress-induced FKBP5 operates in an ultrashort self-regulatory negative feedback loop in control of GR activation. This is demonstrated in CORT control of the CRH gene: overexpression of FKBP5 causes downregulation of GR, which leads to hyperexpression of CRH [16]. The finding explains how changes in FKBP5 expression, either induced by chronic stress and/or (epi)genetically imposed, may predispose for altered GR-mediated negative feedback regulation and thus produce HPA axis dysregulation that may enhance susceptibility to disease [17].

**Figure 1:**
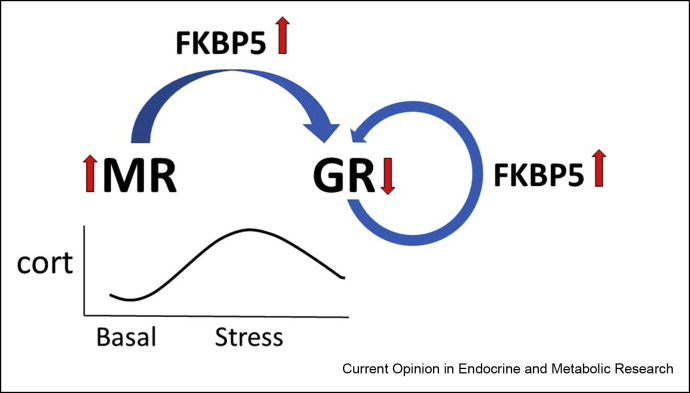
MR-GR and FKBP5. Under basal conditions, MR activation increases the expression of FKBP5, which inhibits the binding CORT to GR; after stress, the rising CORT levels activate additional GR, which further increases FKBP5 synthesis. FKBP5 subsequently decreases GR activity via an ultrashort negative feedback loop [20].

However, under basal conditions—when GR shows little activation—FKBP5 expression appears under the control of co-localized MR [18, 19∗∗, 20∗∗]. For instance, hippocampal CA2 neurons with abundant expression of MR express high basal levels of FKBP5 with little GR activity, while much GR activation is observed following MR blockade or genetic deletion [20]. One would predict, therefore, that high MR expression because of genetic polymorphisms [21] or epigenetically induced by positive (early) life experience [22] would result in increased basal FKBP5 production, which raises the threshold for GR activation in response to an acute stressor. Thus, if co-localized, under basal conditions the degree of MR activation may determine via FKBP5 the tone of GR functionality. As concluded by Jacob Hartmann [20], “this provides additional insights into the molecular mechanism underlying the MR:GR balance hypothesis” (Figure 1).

## Novel hippocampal MR phenotype

Using publicly available single-cell RNA-seq data extracted from the Allen Institute of Brain Sciences Atlas, a detailed map of MR and GR distribution over hippocampal cell types was recently presented [23]. For this purpose, 13 cell types were selected from a database of 77000 cells and approximately 26000 genes including *Nr3c1* and *Nr3c2,* and their downstream target genes encoding a variety of neurotransmitter and neuropeptide receptors. As expected, a high density of MR occurred in excitatory neurons of the hippocampal pyramidal cell fields, particularly neurons of CA2 and dentate gyrus, but surprisingly also to some extent in inhibitory GABAergic neurons. In microglial cells, MR is highly expressed which may explain their pro-inflammatory phenotype in the DOCA-salt model [24] and after chronic stress [25].

Embryonic deletion of MR from hippocampal CA2 neurons resulted in loss of their molecular phenotype as is demonstrated by, e.g., the absence of the regulator of G protein signaling 14 (RGS 14) that otherwise should appear in the mouse at postnatal day 14 [26]. This implies that such MR-knock-out animals display a dysfunctional CA2-based neuronal circuitry as becomes apparent from its uncoupling of the supramammillary network. The various brain MR mutants, including mutants produced by targeted deletions of postnatal MR, all display deficits in social discrimination, reactivity to novelty, decision-making and represent an anxiolytic phenotype [26]. The data led to the conclusion that in CA2 neurons the MR is a so-called “terminal selector” transcription factor critical for cell fate and expression of cell-specific genes not only during development but also during adult life [26].

In another important study, a double hippocampal MR and GR knockout showed not only a loss of CA2 phenotype but also a severe deficit in contextual fear learning. The dentate granular neurons were apoptotic and strongly reduced in number [27], a phenomenon reminiscent to the outcome of adrenalectomy (ADX)-induced neurodegeneration [28]. Accordingly, this finding adds to the growing database on the role of the dentate gyrus in learning and memory processes. Furthermore, hippocampal MR appear required for survival and differentiation of the granular neurons, while GR activation affects the proliferation and migration of the newborn cells [27].

## Novel MR gene targets

Chromatin immunoprecipitation and RNA-seq (ChIP-seq) were used to map genome-wide DNA binding sites for MR and GR in hippocampus [19,29,30], mostly to glucocorticoid response elements (GREs) as (hetero or homo)dimers [27,31]. ADX animals replaced with CORT revealed in hippocampus 918 MR-exclusive sites, 1450 GR-exclusive sites, and 1475 MR-GR binding sites [30]. Onno Meijer's laboratory discovered that most MR-exclusive GRE binding sites were near an AtOH sequence that is targeted by the transcription factor NeuroD, which is thought to potentiate MR binding to GRE. Other DNA sequence motifs were noted for transcription factors such as RFX3, and members of the KLF, EGR, STAT, and FOXO family members that presumably are also needed to guide DNA binding [19,29].

Validation with help of a forebrain MR gene deletion model revealed several selective MR-responsive genes, such as JdP2, a member of the AP-1 family of transcription factors [18]. However, the CORT concentration that enhanced MR binding to the JdP2 GRE and to some other GREs seemed to exceed the limited binding capacity of the MR ligand. A possible explanation is that MR:GR heterodimers are formed which may respond to a larger range of corticosterone concentrations [27,31].

A particularly exciting finding was made in a genome-wide functional analysis of MR and GR binding to DNA in animals exposed to an inescapable stressor [19]. Mifsud and colleagues discovered that the expression of numerous (>50) ciliary genes is under constitutive control of MR. This is important since it would explain how the MR is involved in the neurogenesis of dentate gyrus neurons [19] and perhaps also in the stabilization of cortical dendritic spines underlying learning processes [32].

## GR and the hippocampal memory engram

Recently, Bonapersona et al. [33] presented forebrain c-fos expression with spatial distribution in 3D at 30, 90, and 180 min post-footshock (see also [34]). This 4D approach allowed stress-induced c-fos activity analysis in networks as well as in individual cells. Data extracted from 90 areas clearly demonstrated initial activation of hypothalamic areas, followed by amygdala, prefrontal and hippocampal areas, and finally thalamus, while in some areas such as the basolateral amygdala, c-fos activity changed in caudal-frontal direction over time. The data are available at an interactive web portal (https://utrecht-university.shinyapps.io/brain_after_footshock/).

In hippocampus dentate gyrus, c-fos expression dramatically increased in sparsely distributed single neurons only. Such c-fos labeled cells support the CORT-induced retention of acquired immobility in the forced swim test because of GR-mediated demethylation of CpG sites near the c-fos transcriptional initiation site. Administration of the S-adenosyl methyl donor (SAM) decreased c-fos and impaired memory consolidation [35]. Further studies demonstrated that GR may mediate CORT action via a (non)genomic ERK1/2–MSK1–Elk-1 signaling pathway representing a hypothetical neural substrate underlying memory performance called a memory engram [36]. Another recent study showed that also fear conditioning caused induction of the c-fos dentate memory engram, which was further increased by exogenous CORT. Chemogenetic suppression of engram excitability and c-fos expression attenuated the CORT effect, thereby reinstating contextual memory consolidation at the expense of *fear generalization* [37], the latter being a characteristic symptom of PTSD.

The mechanism underlying glucocorticoid-induced fear memory generalization was also found to depend on the GR-dependent balance between tissue plasminogen activator (tPA) and plasmin activator inhibitor (PAI-1) protein in hippocampus [38]. This tPA:PAI-1 balance is critical for “normal” memory performance, but tPA becomes dysfunctional by excess (exogenous) CORT because of increased synthesis of PAI-1. The tPA deficit abrogates the BDNF-TrkB-Erk^1/2MAPK^ cascade with negative consequences for synaptic function in hippocampus. As a consequence, cue rather than context-dependent memory function is enhanced. Accordingly, hippocampus-dependent memory becomes decontextualized (generalized) suggesting this mechanism also may contribute to PTSD symptomatology [38].

## Salience and executive networks

**Figure 2:**
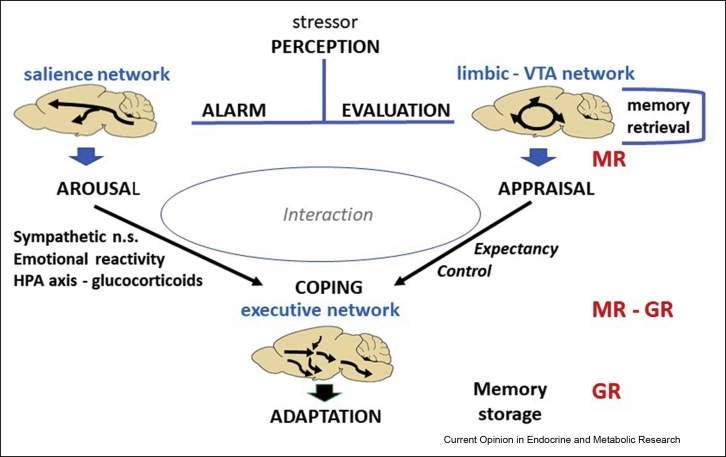
MR-GR and processing of information. If a threat is perceived, an alarm reaction instantaneously develops and the default mode network switches toward the salience neuronal network, while simultaneously the valence of the experience is evaluated [39,48,101]. Arousal is high, and a sympathetic, behavioral (attention, vigilance, and behavioral inhibition), and neuroendocrine stress response develops which interacts with appraisal processes driving the selection of an appropriate coping response. If coping succeeds in fulfilling expectancy, the executive neuronal network takes back control, the stress response is extinguished, the experience is stored in memory, and recovery and behavioral adaptation are promoted: this is the adaptive process, i.e., allostasis at work. If coping fails, uncertainty remains and expectations are frustrated, the stress response is reinforced, eventually the circuits engaged in adaptive information processing may crash (the brain gets stuck [62]), and cognitive control may become overwhelmed by emotional reactivity. If this condition continues allostatic load increases, critical components of the executive network—hippocampus and neuronal ensembles of the mPFC—may become atrophied and dysfunctional, hence driving the breakdown of adaptation toward increased vulnerability to affective and neurodegenerative disorders. CORT modulates the processing of the stressful information from perception to coping and adaptation via MR and/or GR. CORT activation of in particular GR is instrumental in the recruitment of specific neuronal circuits by allocation of energy resources as a function of time and context: under chronic stress conditions, CORT may promote atrophy of hippocampus and mPFC, while supporting the hypertrophy of the emotional amygdala [62]. Cartoon generated after discussion with Pieter Smelik and Bruce McEwen.

Figure 2 shows the flow of information processed from perception (or anticipation) of a stressor toward stress-coping and adaptation. In human fMRI studies, a stressor was found to switch the default mode toward a “salience” network. About an hour later, when CORT activates GR, energy allocation shifts toward an executive network to support cognitive control [39]. In a recent systematic review and meta-analysis of changes in BOLD signal associated with acute stress exposure (31 fMRI studies), Berretz et al. [40] concluded that initial activation of the salience network starts with insular cortex and claustrum, which are crucial for perception and integration of sensory signals. The lentiform nucleus including the globus pallidus and putamen motor neurons are also first, while amygdala and hypothalamus are readily activated as well.

**Figure 3:**
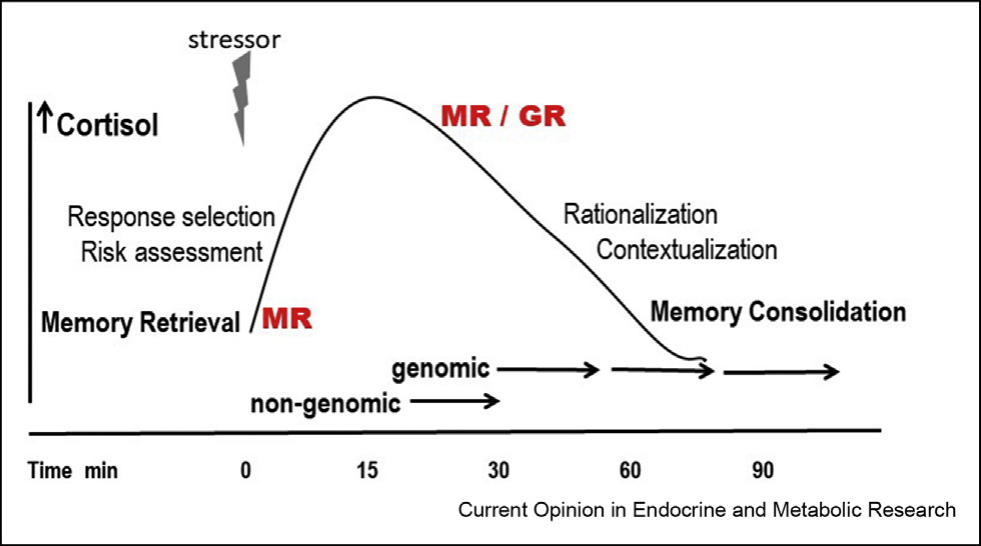
MR-GR and the stress-induced cortisol response. MR is important for the threshold and sensitivity of the stress response system; MR activation facilitates memory retrieval, risk assessment, response selection, and then encoding and learning. Next, with rising CORT levels, GR activation increases and starts the recovery process while promoting rationalization and contextualization of the experience for memory storage in preparation of future encounters. If such an encounter occurs, MR facilitates memory retrieval and so on. Adapted from Ref. [59].

Figure 2 also indicates the CORT receptor type is likely involved in the various phases of information processing. The salience network, i.e., initial alarm and appraisal processes, including risk assessment and cognitive flexibility, are modulated during MR activation as appears from animal studies using MR antagonists and observations with innate or acquired gain or loss of MR function. The MR-dependent memory retrieval and risk assessment are important for the degree of expectancy and therefore critical for the outcome prediction of a selected coping strategy. GR-mediated actions in cooperation with catecholamine signaling rather support the shift toward the executive network, including motivational arousal and memory consolidation (and extinction) in preparation for the future [41,42].

Interestingly, perception of sensory signals (sound, taste, and smell) appears affected by changes in circulating CORT level. Already in the late 1960s, it was noted that Addison patients are impaired in discrimination of sensory stimuli—because of enhanced signal detection—which was normalized with CORT and prednisone rather than mineralocorticoid replacement. This suggests a role for GR in threshold in the detection of sensory signals, but for this conclusion, more research is required. Recently, Obleser et al. [43] demonstrated that the circadian change in circulating CORT level indeed correlates with perception discrimination ability.

## mPFC projectome, coping, and neuroendocrine control

With the recent advance in optogenetic and chemogenetic approaches combined with fiber photometry, the functional anatomy of the medial prefrontal cortex (mPFC) is becoming better understood. Thus, the more pro-active aspects (expectancy and motivation) of cognitive control rather belong to dorsal (dmPFC)/prelimbic neuronal ensembles [44,45], while the ventral (vmPFC)/infralimbic area seems involved in reactive behavior [46, 47, 48]. The studies suggest that excitatory glutamatergic mPFC efferents control neuroendocrine regulation [49], but that this control involves downstream GABA-ergic relays.

Whole forebrain GR deletion from excitatory rather than inhibitory neurons caused loss of a fearful phenotype [50]. However, on the mPFC level only, chronic stress caused resistance to GR-mediated control of inhibitory interneurons. This results in a reduction of excitatory mPFC outflow and a reduced inhibitory control over anxiogenic processe in the amygdala [51,52]. Sex differences were noted, while the effects of early life stress [53] and environmental enrichment [54] also resonate in mPFC functionality. Even the effect of a single prolonged stress experience becomes manifest in a long-lasting reduction of vmPFC activity [55] [56]. This work in progress by the Jim Herman lab using combinations of optogenetic, DREADD and fiber tracing technology reveals the complexity in the control of the E/I balance by CORT.

After having established a critical role of the anteroventral bed nucleus of the stria terminalis (avBNST) in dmPFC control over HPA axis activity and coping style [44,57], Radley's group went on to manipulate optogenetically the avBNST efferents to either the paraventricular nucleus (PVN) or to the ventrolateral periaqueductal grey (vlPAG), while using CORT output linked to passive (inhibitory) avoidance as endpoints, respectively [58]. Briefly, (i) blockade of the GABAergic avBNST input to PVN immediate after learning -mimicking a chronic stress-induced reduction in excitatory dmPFC input- raised via HPA axis stimulation CORT output and the increased CORT concentration then *enhanced* memory retention of the passive avoidance measured 24 hours later. (ii) Stimulation of the avBNST GABAergic projection to the vlPAG directly after learning *impaired* memory retention of passive avoidance behavior, but did not affect adrenal CORT secretion. The data suggest a complementary CORT-dependent and independent mechanism underlying consolidation and retention of fear-motivated inhibitory avoidance. To fully appreciate the complexity of this ingenuous experiment one needs to realize that the test measures, at 24 hours after learning, risk assessment and decision-making between the memory to the electric shock vs the adverse situation of actual light exposure.

Accordingly, this experiment illustrates the cooperation between *top-down* mPFC control directly exerted via the vlPAG motor output and the *bottom-up* CORT control of memory storage of the selected coping style via an action of the hormone mediated by MR and GR in the prefrontal-limbic brain circuitry [59].

## CORT patterns

Diurnal CORT patterns are driven by the suprachiasmatic clock in anticipation of daily and sleep-related events [1]. The circadian rhythm is made up of hourly CORT pulses, which are driven by an adrenal-pituitary feedback loop. Interestingly, the hourly pulses are high enough throughout the day to maintain nuclear localization of the MR, but GR nuclear translocation follows the hourly pulses. This gene pulsing by CORT is necessary for the maintenance of GR responsivity. Accordingly, a flattened CORT rhythm, as in depression and aging, goes along with reduced GR responsivity, e.g., CORT resistance [60].

In the CORT response patterns to repeated stress exposure, Bruce McEwen distinguished at least two different deficits [61,62]. Firstly, lack of adaptation in *CORT peak amplitude* which points to a deficit of MR in the regulation of the threshold, sensitivity, and onset of the stress response. Secondly, lack of adaptation in *turning off the CORT response* which points to a GR-mediated feedback deficit (Figure 3). Indeed, in a peripubertal stress paradigm from postnatal day (pnd) 28 to pnd 42, the two prototypes can be distinguished. Animals with a lack of peak CORT adaptation (re: MR deficiency) were impaired in hippocampal spatial learning [63]. The animals that maintained a prolonged CORT response showed impaired social competence and increased anxiety, a phenotype that could be reversed with the GR antagonist mifepristone [64].

Healthy men show higher stress-induced HPA axis activity than observed in females as can be judged from ACTH, saliva free, and blood total cortisol levels [65]. Also, in a systematic review and meta-analysis, Zorn et al. [66] showed that depressed males have an increased HPA axis response in the Trier Social Stress Test (TSST), which is a psychosocial stressor characterized by socio-evaluative threat and lack of control. The HPA axis response of females to the TSST is, however, attenuated, if compared to the male pattern. Upon remission of the depressive symptoms, the sex difference in HPA axis activity remains [66].

In rodents, the reverse is observed: stress-induced HPA axis activity is generally higher in females than in males with the highest ACTH and corticosterone levels during pro-estrus [67,68]. Why sex differences in stress-induced HPA axis activity are opposite to the human pattern is not known. It could be related to the type of stressor since males and females have generally different coping styles.

## Reset of the stress response system

The GR/progesterone antagonist mifepristone is used for the treatment of inoperable Cushing disease and alleviates psychotic depression symptoms [69]. In animal models, mifepristone ameliorates metabolic disorders [70] and halts alcohol dependency [71]. In models of Alzheimer disease, mifepristone slows down or even reverses cognitive decline and reduces amyloid and tau pathology [72]. Prolonged treatment (three weeks) with mifepristone prevented deficits in motor behavior of the Wobbler rat, a model for amyotrophic lateralis sclerosis (ALS). Mifepristone suppressed in spinal cord glia's a chronic inflammatory feedforward loop, opposed markers for apoptosis, promoted survival pathways, and attenuated glutamate excitotoxicity [73].

While the neurodegenerative models required prolonged treatment with GR antagonist, other animal research suggests that mifepristone can readily reset a dysregulated stress response system. Firstly, brief treatment with mifepristone reversed suppression of dentate gyrus neurogenesis caused by chronic corticosterone administration or chronic unpredictable stress exposure [74]. Secondly, contextual memory deficits in animals exposed as pups to early adversity were ameliorated with peripubertal mifepristone [75]. Thirdly, in the peripubertal stress paradigm, mifepristone not only attenuated aggression if administered in the context of the peripubertal stress paradigm but also at adulthood “out of the stress context”, thus several days before testing aggressiveness [64]. Likewise, a reset of neuroendocrine and behavioral responses occurred with mifepristone several days after the single prolonged stress paradigm [76]. How mifepristone can exert this lasting reset in behavioral performance is unknown but could be related to the upregulation of MR and lasting changes in HPA axis responsivity [77].

More selective glucocorticoid receptor modulators (SGRMs) are currently being developed [78]. Thus, CORT 113176 combines antagonism toward neurodegeneration with agonist anti-inflammatory activity while being more selective than mifepristone in blocking the ALS phenotype in the Wobbler rats [73]. CORT 118335, CORT 122928, and CORT 125134 (relacorilant) reduced alcohol self-administration [71]. CORT 108297 promotes memory consolidation as agonist, while CORT 118335 is a potent GR antagonist that also displays some MR antagonism [79]. CORT 125329 is a selective GR antagonist that is promising for the treatment of metabolic disease [80]. With help of the Allen Brain atlas, the cellular distribution and co-localization of numerous coregulators with MR and GR have been mapped, which may guide the application of cell and tissue-specific SGRMs [79]. Selective mineralocorticoid receptor mediators (SMRMs) are currently being developed [81].

## Conclusion

Progress in understanding of MR and GR-mediated actions in neuroendocrine regulation and psychiatric etiopathologies was briefly sketched in the preceding sections. Thus, in hippocampus and likely elsewhere, FKBP5 seems an important determinant in fine-tuning MR-GR activation [20]. MR was indicated as “terminal selector” of the hippocampal CA2 neuronal phenotype [26]. Functional characterization of genomic MR-GR binding sites provides numerous new leads for further research [18,19]. Evidence was strengthened for a GR-dependent hippocampal dentate gyrus memory engram [37]. On a more integrated level, the mPFC projectome was further dissected in its various circuits underlying stress-coping, memory performance, and neuroendocrine regulation [58]. HPA-axis patterns may serve as potential biomarkers of vulnerability or resilience to stress-related psychopathology [61, 62, 63, 64∗∗], but these patterns display a remarkable sex difference [65,67].

## Perspectives

Firstly, CORT actions are primarily concerned with energy metabolism. Hence, one could view MR-mediated actions in risk assessment and response selection as an economic strategy to deal with an (anticipated) threat or reward with less cost. GRs function would be then to allocate energy resources to brain regions involved in emotional, motivational, and cognitive processes to regain control and adapt. The late Bruce McEwen defined this adaptive process as allostasis and the associated energy expenditure as allostatic load [82]. Attempts are underway to exploit an allostatic load index (ALI) as a measure of physical and mental fitness [83,84]. In this respect, the action of glucocorticoids in mitochondria during psychosocial stress adaptation is a new and challenging field of science [85].

Secondly, regarding resilience to stress-related disorders, it is becoming more and more accepted that lifestyle patterns, exercise, and social support may help to “take back control” and “teach” CORT secretion patterns that favor resilience [86,87].

Thirdly, the treatment of immune and inflammatory disorders with synthetic glucocorticoids is limited because of the unwanted side effect on, e.g., energy metabolism and suppression of HPA axis activity. New SGRMs and SMRMs may be useful because of their increased selectivity under different psychosocial stress contexts. Moreover, these new compounds can rebalance MR and GR-mediated actions which opens the possibility to reset stress-induced CORT patterns critical for resilience [77,88].

Finally, a major challenge is to better understand how hormones, such as CORT, that have in every cell different actions, can coordinate cell, body and brain function during circadian events and in response to (or anticipation of) stressors, and integrate these responses over time in different contexts with one obvious goal: to facilitate the adaptive process. Selye distinguished antagonistic adaptive hormones for that purpose, an action that can be exerted with today’s wisdom by CORT via GR and the phylogenetic older MR . The question is today how MR and GR-mediate *bottom-up* in complementary manner permissive, suppressive, stimulatory, and preparative actions of CORT in various tissues, including the limbic forebrain, that exerts *top-down* cognitive control over mind, brain, and body [4].

To address such a question, one can envision a computation strategy that would involve bioinformatics to predict resilience to stress-related neuropsychiatric symptoms based on genetic MR-GR variants and epigenetic cell-specific transcriptome signatures [89]. Such an approach would offer challenging opportunities that reinforce Tausk's definition of Endocrinology as a concept, approach, or method to unravel “how signals coordinate the processes in cells, tissues, and organs.”

## Conflict of interest statement

ER de Kloet owns stock of Corcept Therapeutics.

## Footnotes

1 Marius Tausk (1902 – 1980) professor of Endocrinology, University of Utrecht, The Netherlands and Director of Organon, on the occasion of the opening symposium of the Netherlands Endocrine Society, May 10, 1947.
